# Airway Basal Stem Cells Inflammatory Alterations in COVID‐19 and Mitigation by Mesenchymal Stem Cells

**DOI:** 10.1111/cpr.13812

**Published:** 2025-01-26

**Authors:** Sheng Du, Jing Jin, Chunli Tang, Zhuquan Su, Lulin Wang, Xinyuan Chen, Mengni Zhang, Yiping Zhu, Jiaojiao Wang, Chunrong Ju, Xinyu Song, Shiyue Li

**Affiliations:** ^1^ State Key Laboratory of Respiratory Disease, National Clinical Research Center for Respiratory Disease, National Center for Respiratory Medicine Guangzhou Institute of Respiratory Health, the First Affiliated Hospital of Guangzhou Medical University Guangzhou Guangdong China; ^2^ Guangzhou National Laboratory Guangzhou China

**Keywords:** airway basal stem cells, COVID‐19, fibrosis, goblet cell hyperplasia, inflammation, mesenchymal stem cells, post‐acute sequelae of COVID‐19

## Abstract

SARS‐CoV‐2 infection and the resultant COVID‐19 pneumonia cause significant damage to the airway and lung epithelium. This damage manifests as mucus hypersecretion, pulmonary inflammation and fibrosis, which often lead to long‐term complications collectively referred to as long COVID or post‐acute sequelae of COVID‐19 (PASC). The airway epithelium, as the first line of defence against respiratory pathogens, depends on airway basal stem cells (BSCs) for regeneration. Alterations in BSCs are associated with impaired epithelial repair and may contribute to the respiratory complications observed in PASC. Given the critical role of BSCs in maintaining epithelial integrity, understanding their alterations in COVID‐19 is essential for developing effective therapeutic strategies. This study investigates the intrinsic properties of BSCs derived from COVID‐19 patients and evaluates the modulatory effects of mesenchymal stem cells (MSCs). Through a combination of functional assessments and transcriptomic profiling, we identified key phenotypic and molecular deviations in COVID‐19 patient‐derived BSCs, including goblet cell hyperplasia, inflammation and fibrosis, which may underlie their contribution to PASC. Notably, MSC co‐culture significantly mitigated these adverse effects, potentially through modulation of the interferon signalling pathway. This is the first study to isolate BSCs from COVID‐19 patients in the Chinese population and establish a COVID‐19 BSC‐based xenograft model. Our findings reveal critical insights into the role of BSCs in epithelial repair and their inflammatory alterations in COVID‐19 pathology, with potential relevance to PASC and virus‐induced respiratory sequelae. Additionally, our study highlights MSC‐based therapies as a promising strategy to address respiratory sequelae and persistent symptoms.

## Graphical abstract

Airway basal stem cells derived from COVID‐19 patients demonstrated pronounced goblet cell hyperplasia, inflammatory responses and tendencies toward fibrosis. Co‐culture with mesenchymal stem cells (MSCs) notably reduced these pathological changes, likely due to MSCs' regulatory effects on the interferon signalling pathway.

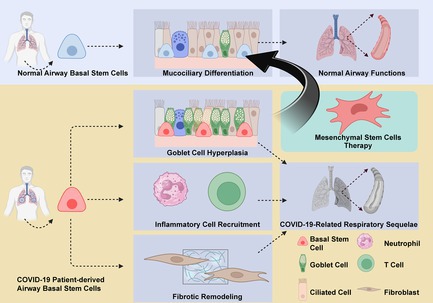

## 1 Introduction

The Coronavirus Disease 2019 (COVID‐19) pandemic, caused by Severe Acute Respiratory Syndrome Coronavirus 2 (SARS‐CoV‐2), has posed significant challenges in understanding and managing respiratory viral infections [1]. Central to COVID‐19 pathology is the disruption of the respiratory epithelium by SARS‐CoV‐2, leading to severe clinical outcomes [2, 3, 4, 5]. Clinical symptoms include excessive airway mucus secretion [6, 7], severe pulmonary inflammation [8, 9, 10, 11, 12] and extensive lung fibrosis [13, 14], which impair lung function and contribute to long‐term complications in affected patients referred to as long COVID or post‐acute sequelae of COVID‐19 (PASC) [3, 4].

Airway basal stem cells (BSCs) are a vital component of the airway epithelium, serving as reservoirs for tissue regeneration and repair. These stem cells, residing just above the basement membrane, constitute approximately 6%–30% of the airway epithelial cells, depending on the region and health state. Identified by markers such as transformation related protein 63 (P63) and keratin 5 (KRT5), BSCs play a crucial role in maintaining epithelial homeostasis and responding to injury and disease [15, 16]. In COVID‐19‐affected airways, there is notable mucus hypersecretion, widespread inflammatory cell infiltration and fibrotic transformation associated with respiratory epithelium damage [10, 11]. Studies have shown that BSCs proliferate in COVID‐19 patients, highlighting their critical role in the disease's pathology [17]. Investigating the fundamental characteristics and behaviours of BSCs is essential for developing therapeutic strategies to mitigate the adverse effects of COVID‐19.

The exploration of BSCs in the context of COVID‐19 faces significant obstacles. First, the direct isolation of BSCs from COVID‐19 patients is challenging. Consequently, researchers largely depend on single‐cell sequencing and immunofluorescent staining of lung sections, along with in vitro models simulating SARS‐CoV‐2 infection. While these techniques are essential, they may not fully capture the intricate behaviours and dynamics of BSCs under authentic disease conditions. Second, developing xenograft models using BSCs from COVID‐19 patients presents significant difficulties. Although BSC‐based xenografts are valuable for studying respiratory diseases like chronic obstructive pulmonary disease (COPD) and idiopathic pulmonary fibrosis (IPF), adapting these models for COVID‐19‐specific research remains unfulfilled [18, 19]. These xenograft models are crucial for elucidating interactions between BSCs and other cellular entities and for translating laboratory findings into clinical applications [20]. These challenges highlight the need for advanced methodologies to explore the complex roles and therapeutic potential of BSCs in COVID‐19 and PASC.

Despite progress in COVID‐19 treatment, particularly in the development of antiviral drugs and vaccines, the options targeting the intense inflammation caused by the disease remain limited. Mesenchymal stem cells (MSCs) have shown anti‐inflammatory properties, making them a promising therapeutic option [21]. Their application in COVID‐19 clinical trials has demonstrated potential in mitigating virus‐induced airway damage, managing ARDS, reducing mortality and enhancing recovery [21]. Nonetheless, the precise mechanisms by which MSCs influence COVID‐19 BSCs—including their ability to modulate the inflammatory response and promote the repair and regeneration of the airway epithelium—require further investigation.

This study marks the first successful isolation of BSCs from COVID‐19 patients in the Chinese population and the first establishment of a xenograft model derived from these cells. Utilising the largest patient cohort to date, we examined the intrinsic properties of COVID‐19 BSCs through in vitro and in vivo differentiation assays, augmented by epithelial–immune/mesenchymal cell co‐culture experiments. Through RNA sequencing, we mapped the cellular alterations associated with COVID‐19, providing a comprehensive understanding of the disease's impact at the molecular level. Our data indicate that BSCs in the epithelia of COVID‐19 patients exhibit enduring pathological alterations related to PASC, suggesting their impaired function may contribute to the disease's progression by influencing local tissue dynamics and interactions with surrounding cell types. Building on these findings, we explored the therapeutic influence of MSCs on these altered BSCs through in vitro and in vivo co‐culture assays. Our results demonstrate that MSCs can mitigate the pathological phenotype of COVID‐19 BSCs, providing promising evidence of their potential to restore healthy epithelial functions and promote recovery in COVID‐19 patients.

## 2 Methods

### 2.1 Patients and Sampling Method

Between December 2022 and May 2023, patients at the First Affiliated Hospital of Guangzhou Medical University who required bronchoscopy were recruited for this study. Participants clinically diagnosed with COVID‐19, due to Omicron variant infection, constituted the COVID‐19 group, while those free from pulmonary infections were categorised as the control group. During bronchoscopies, airway brushing samples were collected from the 4th to 6th level airways. These samples served for comparative analysis to assess COVID‐19's effects on the airway epithelium. The study received ethical approval from the hospital's Ethics Committee (Ethics ID: 2022138), aligning with the Declaration of Helsinki. Informed consent was obtained from all participants.

### 2.2 BSCs Isolation and Culture Method

Brushing samples from airways were promptly submerged in a patented culture medium (ZL 202210011084.3, Guangzhou Institute of Respiratory Health, China) uniquely designed to support only BSCs, optimising their preservation. The samples were then carefully pipetted to detach and resuspend the cells. The cells were then directly plated onto 6‐ or 12‐well culture plates at an optimal density that promotes cell attachment and expansion. Cultivation was carried out at 37°C in a humidified atmosphere containing 5% CO₂. Isolated BSCs from COVID‐19 group were confirmed SARS‐CoV‐2 RNA‐free.

### 2.3 Clonogenic Assay

The clonogenic assay was employed to evaluate the proliferation capacity of BSCs. Following trypsinisation, filtration was performed through a 40 μm mesh to ensure a single‐cell suspension. BSCs at Passage 5 were seeded at a density of 1000 cells per well in 12‐well culture plates, with triplicate wells for each experimental condition to ensure consistency and reliability. The cells were cultured at 37°C in a 5% CO₂ humidified atmosphere for 7 days. To support optimal cell growth, the culture medium was refreshed mid‐way through the incubation period. After 7 days, the colonies were carefully washed with PBS, fixed with 4% paraformaldehyde (BL539A, Biosharp, China) and stained with 0.5% crystal violet for visualisation. Colonies containing more than 10 cells were identified and quantified using a light microscope or manual colony counter.

### 2.4 Differentiation Assay

For in vitro assay, BSCs were seeded onto 6.5 mm Transwell inserts featuring a 0.4 μm pore polyester membrane at a density of 2.4 × 10 [6] viable cells/cm [2]. Initially, BSCs underwent a growth phase for 3 days in PneumaCult‐Ex Plus medium (05040, STEMCELL, Canada). Subsequently, to initiate differentiation, the culture medium in the lower chamber was switched to PneumaCult‐ALI (05001, STEMCELL, Canada), promoting air–liquid interface (ALI) conditions conducive to BSCs maturation. This differentiation process was sustained for a further 14–21 days to ensure full development of the epithelial architecture. Upon maturation, samples were embedded in paraffin, sectioned and assessed using IF staining. Simultaneously, cellular samples were harvested and preserved in TransZol Up (ET111‐01‐V2, TransGen, China) reagent for RNA isolation purposes.

For in vivo assay, 2 × 10 [6] cells were suspended in Dulbecco's Modified Eagle Medium (DMEM) and mixed with Matrigel in a 1:1 ratio. This 100 μL cell‐Matrigel mixture was then subcutaneously injected into immunodeficient NCG mice (Gempharmatech, China). The male mice aged 4–5 weeks were used for all experiments and were killed at 4 weeks post‐injection. Tissue samples were embedded in paraffin, sectioned and assessed using IHC/IF. All in vivo experimental procedures were performed in strict accordance with the guidelines of the IACUC and received ethical approval (Ethics ID: B202302‐11) from the Guangdong Medical Laboratory Animal Center.

### 2.5 Co‐Culture Assay

#### 2.5.1 In Vitro BSCs–MSC Co‐Culture

BSCs from the COVID‐19 group were cultured on Transwell inserts featuring a 0.4 μm pore polyester membrane to induce ALI differentiation. At Day 8 of differentiation, the transwell inserts were transferred to the lower chamber of a 24‐well plate pre‐seeded with 50,000 human bone marrow derived MSCs (Cellgenes, China) [22, 23] per well to continue ALI culture. The differentiation medium was changed every 2–3 days, with the co‐culture process extending up to Day 14. On Day 14 of co‐culture, cells were examined under an inverted microscope to assess whether they exhibited differentiation characteristics. Subsequently, the upper chamber samples were collected for RNA extraction using TransZol Up, followed by transcriptome sequencing analysis.

#### 2.5.2 In Vivo BSCs and MSC Co‐Culture

The study involved NCG mice allocated to three groups to evaluate the interactions between BSCs and MSCs. For the COVID‐19 group, mice received a subcutaneous injection of 1 × 10 [6] COVID‐19 BSCs per sites, using a DMEM (11965092, Gibco, USA) and Matrigel (356234, Corning, USA) suspension mixed at a 1:1 ratio of 100 μL. In the MSC (i.v.) group, mice underwent subcutaneous injections of 1 × 10 [6] COVID‐19 BSCs per site, using a DMEM and Matrigel suspension 100 μL mixed at a 1:1 ratio. After 7 days, these mice were given an intravenous injection of 1 × 10 [6] MSCs in a 200‐μL PBS solution via the tail vein. The MSC (s.c.) group consisted of subcutaneous injections of a combined suspension of 5 × 10 [5] MSCs and 1 × 10 [6] COVID‐19 BSCs per site. 100 μL suspension was prepared in a 1:1 ratio of DMEM and Matrigel.

#### 2.5.3 In Vitro BSCs and HFL‐1 Cells Co‐Culture

This assay was carried out following the previous description with slight modification [18]. In brief, human fetal lung fibroblast 1 (HFL‐1) cells (CL‐0106, Procell, China) were propagated to 70% confluency in preparation for co‐culture. Upon reaching density, HFL‐1 cells were harvested, and 50,000 cells were seeded into each well of a 24‐well plate. These cells were then incubated for 24 h. Subsequently, 25,000 BSCs were introduced to the wells containing pre‐cultured HFL‐1 cells. The co‐culture was sustained with the culture medium refreshed bi‐daily to facilitate BSCs and HFL‐1 cell interaction. After 7 days, the co‐culture samples were fixed for immunofluorescence (IF) staining to evaluate cellular responses and interactions.

#### 2.5.4 Immunostaining and Histological Analysis

Histological and immunostaining techniques, including haematoxylin and eosin (H&E) staining, were conducted following routine laboratory methods. Cultured cells or tissues were fixed in 4% paraformaldehyde for either direct staining or subsequent paraffin embedding and sectioning. Cells or sections underwent permeabilisation with 0.1% Triton X‐100 in PBS enriched with 5% bovine serum albumin, followed by incubation with the detailed primary antibodies as specified subsequently: rabbit monoclonal Ly6G antibody (ab238132, Abcam, UK), mouse monoclonal Tubulin antibody (T6793, Sigma‐Aldrich, USA), rabbit monoclonal human HLA‐A antibody (ab52922, Abcam, UK), rabbit monoclonal E‐cadherin (E‐cad) antibody (3195, CST, USA), rabbit monoclonal mucin 5AC antibody (ab198294, Abcam, UK), rabbit monoclonal human cytokeratin 5 antibody (ab52635, Abcam, UK), mouse monoclonal p63 antibody (ab735, Abcam, UK) and mouse monoclonal alpha smooth muscle actin antibody (ab7817, Abcam, UK). Secondary antibodies used here are Alexa Fluor 488 goat anti‐mouse IgG (ab150113, Abcam, UK), Alexa Fluor 594 goat anti‐rabbit IgG (ab150080, Abcam, UK), Alexa Fluor 488 goat anti‐rabbit IgG (ab150077, Abcam, UK) and Alexa Fluor 594 goat anti‐mouse IgG (ab150116, Abcam, UK) and goat anti‐rabbit IgG, peroxidase conjugated, H + L (BL003A, Biosharp, China) and DAB Chromogenic Kit (G1212, Servicebio, China) to visualise the bound primary antibodies. IF images were captured using Leica DM6 upright digital microscope and Leica DMi8 Inverted Microscopes (Leica, Germany). IHC and H&E images were captured by PRECICE 500B (UNIC, China). Fluorescence images were quantitatively analysed using ImageJ software.

#### 2.5.5 Quantifying Inflammation Infiltration in Xenografts

Based on a method adapted from [Cell, 2020;181 (4):848–864] [19], neutrophil infiltration in xenograft tissue sections was assessed using anti‐murine Ly6G IHC staining. Infiltration levels were categorised into four distinct groups: (1) ‘None’: No detectable neutrophils within the cyst area. (2) ‘Low’: Scattered neutrophils covering less than 25% of the cyst area. (3) ‘Medium’: Moderate accumulation of neutrophils, covering 25%–50% of the cyst area. (4) ‘High’: Dense neutrophil presence, exceeding 50% of the cyst area. The infiltration ratio was calculated using the percentage of cysts classified as ‘Low’, ‘Medium’ and ‘High’ relative to the total cysts observed per tissue section.

#### 2.5.6 Quantifying Fibrotic Remodelling in Xenografts

Fibrotic remodelling in xenograft tissue sections was assessed using IF staining for α‐smooth muscle actin (α‐SMA) as a marker of myofibroblast activation. The extent of fibrosis was quantified by calculating the proportion of α‐SMA‐positive area relative to the combined stained area of E‐cad within each tissue section.

#### 2.5.7 Flowcytometry Analysis

To explore the immunomodulatory effects of ALI conditioned medium on peripheral blood mononuclear cells (PBMCs) from healthy volunteers, we first collected the conditioned medium from ALI cultures and filtered it through a 22‐μm filter to remove cellular debris. PBMCs were then isolated from peripheral blood of healthy volunteers using the Ficoll Paque Plus (17‐1440‐02, Cytiva, USA) density gradient method, and 500 μL of this ALI conditioned medium was mixed with an equal volume of PBMC culture medium containing 5 × 10 [5] PBMCs. The resultant mixture, in addition to 50% ALI conditioned medium, contained 10 U/mL of interleukin‐2, human (11147528001, Roche, Switzerland), 10% heat‐inactivated fetal bovine serum (10099‐141, Gibco, USA), a 1× cell activation cocktail (with Brefeldin A) (423303, Biolegend, USA) and 40% RPMI (11875093, Gibco, USA) medium to foster the activation of PBMCs, followed by incubation for 24 h. Before flow cytometry analysis, PBMCs underwent staining for extracellular markers for 30 min, then proceeded to fixation and permeabilisation using Fixation/Permeabilisation Solution Kit (554714, BD biosciences, USA) for 15 min, with subsequent intracellular staining for 30 min. Each of these steps was performed on ice and in the dark. Subsequently, flow cytometry was utilised using Fortessa X‐20 (BD‐LSR, USA) to evaluate the PBMCs for changes in cell activation and the expression of specific markers. Antibodies or reagents used in this experiments include LIVE/DEAD Fixable Aqua Dead Cell Stain Kit (L34957, Invitrogen, UK), anti‐CD3‐FITC (555339, BD Pharmingen, USA), APC‐H7 Mouse Anti‐Human CD4 (560158, BD Pharmingen, USA), PerCP‐Cy5.5 Mouse Anti‐Human CD8 (565310, BD Pharmingen, USA), Brilliant Violet 71 anti‐human CD45RA Antibody (304138, BioLegend, USA), anti‐TNF PE‐Cy7 (557647, BD Pharmingen, USA) and anti‐IFN γ‐APC (557647, BD Pharmingen, USA).

#### 2.5.8 RNA Extraction, Library Construction and Sequencing

Total RNA was isolated from BSCs and related ALI samples following the guidelines provided by the TransZol Up (ET111‐01‐V2, TransGen, China) reagent. RNA quality was assessed on an Agilent 2100 Bioanalyzer (Agilent, USA) and checked using RNase‐free agarose gel electrophoresis. After total RNA was extracted, eukaryotic mRNA was enriched by Oligo(dT) beads. Then, the enriched mRNA was fragmented into short fragments using fragmentation buffer and reversely transcribed into cDNA by using NEBNext Ultra RNA Library Prep Kit for Illumina (7530, NEB, USA). The purified double‐stranded cDNA fragments were end‐repaired, and A base was added and ligated to Illumina sequencing adapters. The ligation reaction was purified with the AMPure XP Beads (1.0×). Polymerase chain reaction amplification was performed, and the resulting cDNA library was sequenced using Illumina Novaseq6000 by Gene Denovo Biotechnology Co. (Guangzhou, China).

#### 2.5.9 Bioinformatics Analysis

Reads obtained from the sequencing machines include raw reads containing adapters or low‐quality bases which will affect the following assembly and analysis. Thus, to get high‐quality clean reads, reads were further filtered by fastp (version 0.18.0). Short read alignment tool Bowtie2 (version 2.2.8) was used for mapping reads to ribosome RNA (rRNA) database. The rRNA‐mapped reads then will be removed. The remaining clean reads were further used in assembly and gene abundance calculation. An index of the reference genome was built, and paired‐end clean reads were mapped to the reference genome using HISAT2.2.4 and other parameters set as a default. The mapped reads of each sample were assembled by using StringTie v1.3.1 in a reference‐based approach. For each transcription region, a FPKM (fragment per kilobase of transcript per million mapped reads) value was calculated to quantify its expression abundance and variations, using RSEM software. RNAs differential expression analysis and PCA analysis were performed by DESeq2 package in R (version 4.3.0) between two different groups. The heatmap analysis of different samples was performed using pheatmap package (http://cran.rproject.org/web/packages/pheatmap/index.html) in R (version 4.3.0). The enrichment analysis was performed using clusterProfiler [24].

#### 2.5.10 Statistical Analysis and Visualisation

Statistical analyses and graph generation were conducted using GraphPad Prism 9 software, along with R packages ggplot2 and clusterProfiler for detailed visualisation. For data following a normal distribution, comparisons were made using Student's *t* test for two groups or one‐way analysis of variance (ANOVA) for three groups, with subsequent Tukey's multiple comparisons test to pinpoint specific group differences when ANOVA indicated significant disparities. Non‐normally distributed data were analysed using non‐parametric methods, including the Mann–Whitney *U* test for comparisons between two groups and the Kruskal–Wallis *H* test for multiple groups, with Dunn's multiple comparison test applied to identify differences when the Kruskal–Wallis test was significant. Statistical significance was denoted as follows: ‘ns’ for not significant (*p* ≥ 0.05), ‘*’for *p* < 0.05, ‘**’ for *p* < 0.01, ‘***’ for *p* < 0.001 and ‘****’ for *p* < 0.0001, with these annotations used in the presentation of graphical data.

## 3 Results

### 3.1 Alterations in Goblet Cell Phenotype in COVID‐19 BSCs

**FIGURE 1:**
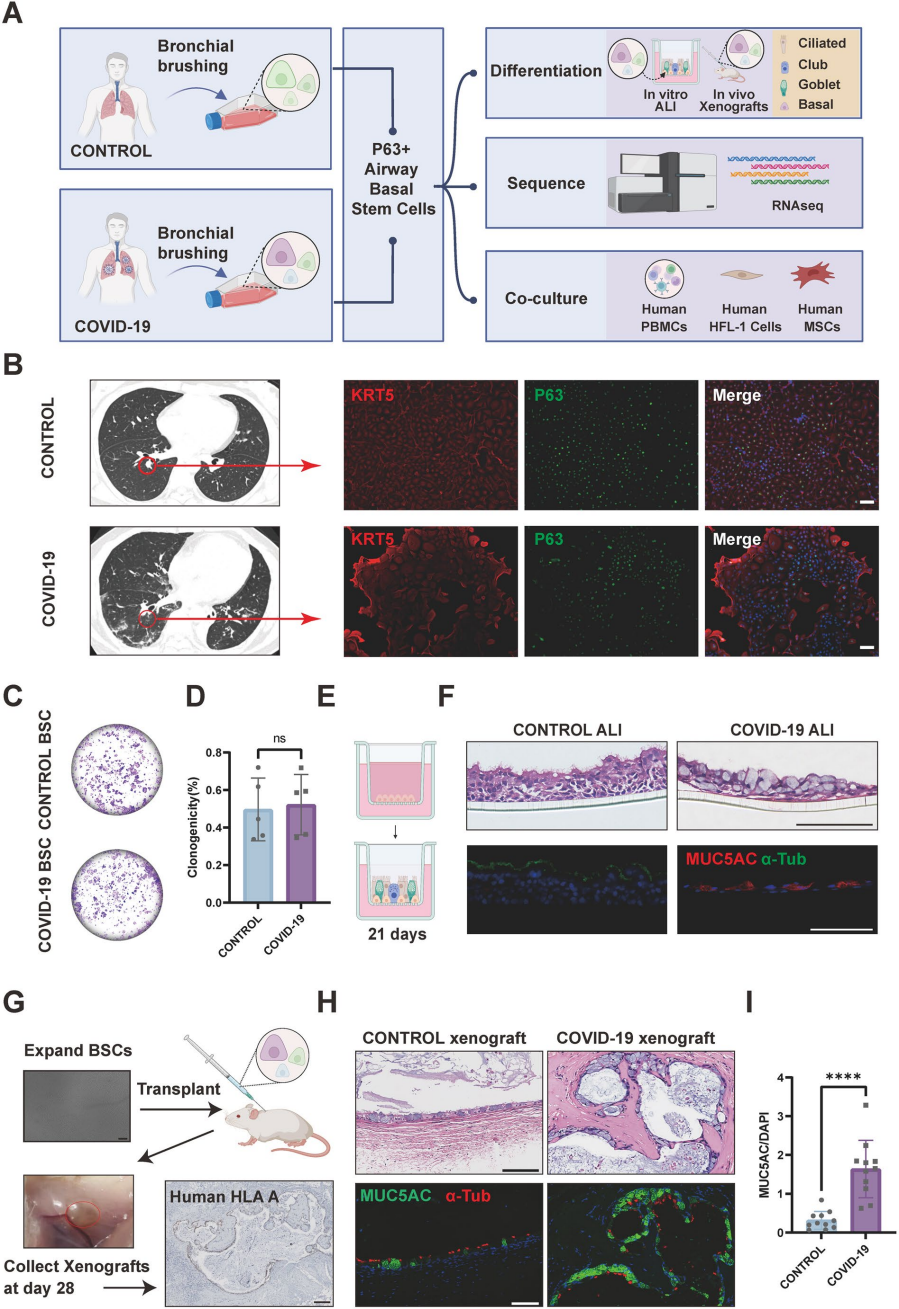
Goblet cell hyperplasia evident in COVID‐19 BSCs. (A) Schematic of BSCs derivation and subsequent functional studies. (B) Immunofluorescence (IF) of KRT5 (red) and P63 (green) for epithelial stem cells from control individual and COVID‐19 patients. Scale bar, 100 μm. (C) Crystal violet stained on colonies from initial 1000 seeded cells from each of control and COVID‐19 BSCs. (D) Quantification of clonogenicity shows the colony formation rate from initially 1000 plated cells, comparing control to COVID‐19 BSCs at Passage 5. Data are represented as the mean ± SD. *n* = 5, unpaired, two‐tailed Student's *t* test, ns = not significant. (E) Diagram illustrating the differentiation of BSCs into airway epithelium via the air‐liquid interface (ALI) method. (F) Top: Haematoxylin–eosin (H&E) staining of control and COVID‐19 ALI. Scale bar, 100 μm. Bottom: IF of MUC5AC (red) and α‐Tub (green) imaging for control and COVID‐19 ALI. Scale bar, 100 μm. (G) The schematic depicts the subcutaneous transplantation of BSCs into immunodeficient NCG mice. Nodules formed by Day 28 were processed for histology and immunostaining. The polarised epithelial structures within these nodules specifically reacted with antibodies against human HLA‐A. Top left: Bright field image of expanded BSCs. Scale bar, 100 μm. Bottom right: Immunohistochemistry (IHC) of human HLA‐A (brown) for BSCs xenograft. Scale bar, 200 μm. (H) Top: H&E staining of control and COVID‐19 xenografts. Scale bar, 100 μm. Bottom: IF of MUC5AC (green) and α‐Tub (red) imaging for control and COVID‐19 xenografts. Scale bar, 100 μm. (I) Quantification of MUC5AC+ cells from control and COVID‐19 xenografts epithelial cysts. Data are represented as the mean ± SD. *n* = 11, unpaired, two‐tailed Student's *t* test, ****p < 0.0001.

In this study, we evaluated the intrinsic properties of airway BSCs derived from COVID‐19 patients compared to those from control individuals (Figure 1A; Tables S1 and S2). Airway brushings were obtained from participants and cultured using an advanced feeder‐free system specifically designed to support the growth of BSCs, allowing them to undergo at least 10 passages. For the COVID‐19 group, initial collections from approximately 60 patients underwent stringent quality control to exclude bacterial or fungal contamination, resulting in 11 high‐quality samples for downstream analysis. In contrast, control group samples exhibited minimal contamination, with all collected specimens successfully processed. BSCs from both COVID‐19 and control groups showed co‐expression of the airway progenitor markers KRT5 and P63 (Figure 1B) and demonstrated similar clonogenic potential of 30%–70% at Passage 5 (Figure 1C,D). To discern the impact of COVID‐19 on BSCs differentiation, we employed an in vitro ALI differentiation protocol (Figure 1E). Control BSCs predominantly differentiated into ciliated epithelium, as evidenced by α‐tubulin staining. Conversely, BSCs from COVID‐19 patients exhibited a proclivity for differentiation into goblet cell‐enriched epithelium, predominantly expressing MUC5AC, indicative of goblet cell hyperplasia (Figure 1F). For in vivo analyses, we transplanted BSCs into highly immunodeficient NCG (NOD/ShiLtJGpt‐Prkdc^em26Cd52^Il2rg^em26Cd22^/Gpt) mice. For each transplantation, 2 million BSCs, combined with 50% Matrigel, were administered subcutaneously. Examination of xenograft nodules 28 days post‐injection revealed that both groups of BSCs could form xenografts containing epithelial cysts. Notably, human‐specific HLA‐A expression confirmed the human origin of these epithelial cysts (Figure 1G). Control BSCs‐derived epithelial cysts mirrored the architecture of pseudostratified ciliated columnar epithelium, characterised by the presence of cilia and sparse goblet cells (Figures 1H and S1A). In contrast, epithelial cysts derived from COVID‐19 BSCs were predominantly composed of densely packed goblet cell, mirroring the in vitro differentiation patterns (Figures 1H and S1A). Quantitative analysis of goblet cell prevalence in xenografts, utilising the ratio of MUC5AC‐positive area to DAPI‐stained area in epithelial cysts, revealed a significant increase in goblet cell metaplasia in the COVID‐19 group compared to controls (Figure 1I). In our pursuit to elucidate the differentiation trajectories of airway epithelium, we scrutinised gene expression profiles from both control and COVID‐19 ALI. Our analyses revealed elevated expression of ACE2, the primary receptor for SARS‐CoV‐2 entry, in COVID‐19 ALI. Additionally, we observed a pronounced upregulation of a suite of genes implicated in goblet cell identity, notably MUC5AC, MUC5B, SCGB1A1, SCGB3A1, as well as transcription factors including FOXA3, SPDEF and AGR2, predominantly in the COVID‐19 ALI. Conversely, genes pivotal to the development and function of mature cilia, such as TPPP3, FOXJ1 and CDC20B, were preferentially expressed in the control ALI (Figure S1C). The difference in gene expression patterns provides molecular evidence of COVID‐19's unique influence on the differentiation of BSCs, propelling a shift toward goblet cell metaplasia, observable both under transcription levels and within living organisms. These findings bear clinical relevance, as they parallel the heightened mucus production observed in patients encountering COVID‐19, hinting at the underlying pathological alterations at the cellular level.

### 3.2 Inflammation Dynamics Within COVID‐19 BSCs

**FIGURE 2:**
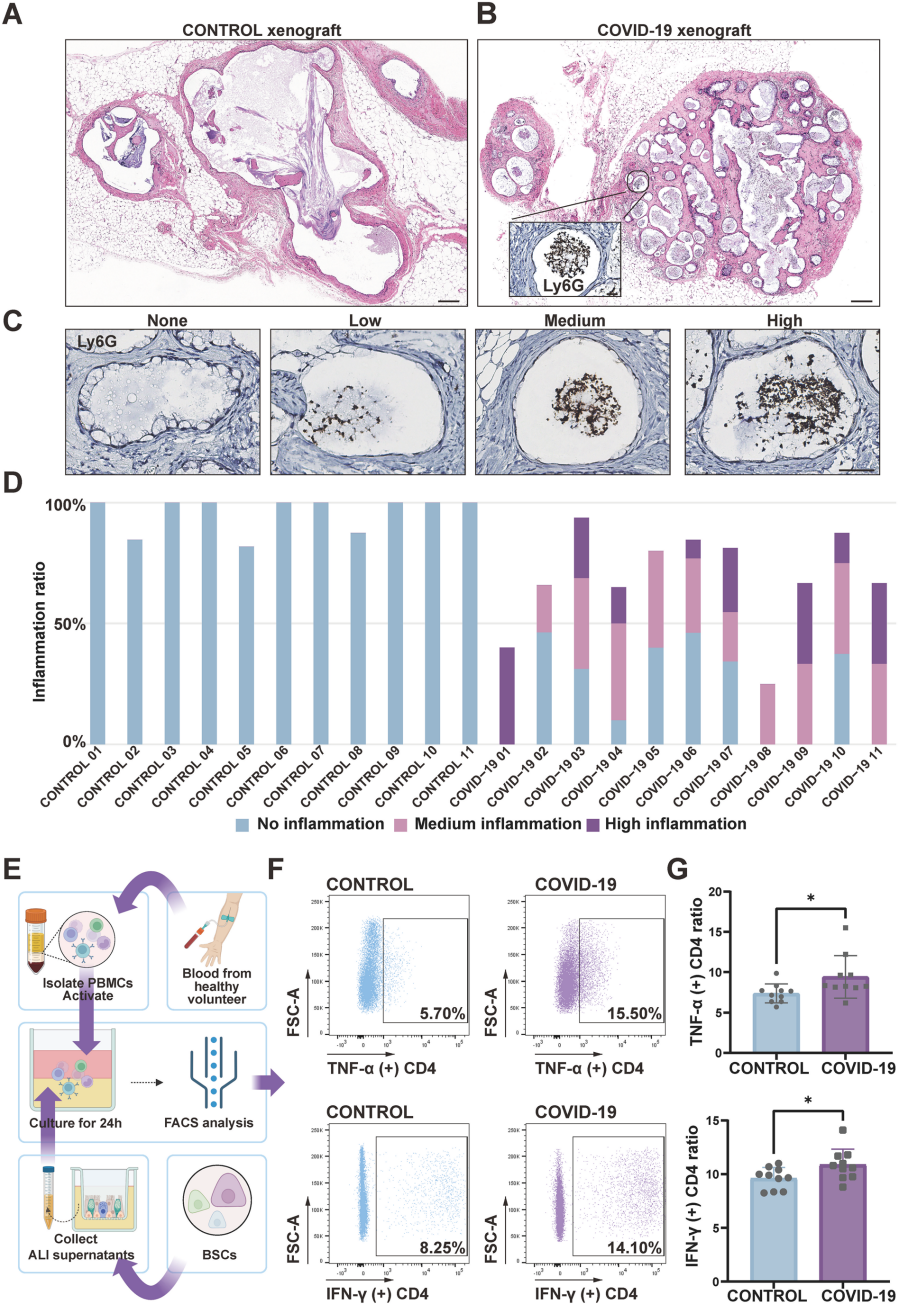
Inflammation dynamics of COVID‐19 BSCs. (A) H&E staining of control xenograft. Scale bar, 200 μm. (B) H&E staining of COVID‐19 xenograft. Scale bar, 200 μm. Insets: IHC of Ly6G for COVID‐19 xenograft. Scale bar, 50 μm. (C) IHC of Ly6G+ leukocyte infiltration degrees within epithelial cysts. Scale bar, 50 μm. (D) Histogram of the quantification of leukocyte infiltration in xenografts from 11 control individuals and 11 COVID‐19 patients. (E) Schematic representation of the experimental workflow for FACS analysis involving PBMCs and ALI supernatants. (F) FACS profiling of TNF‐α (+) CD4 ratio, IFN‐γ (+) CD4 ratio from control or COVID‐19 ALI condition medium stimulated PBMCs. (G) Quantification of TNF‐α (+) CD4 ratio, IFN‐γ (+) CD4 ratio from control or COVID‐19 ALI condition medium stimulated PBMCs. Data are represented as the mean ± SD. *n* = 10, unpaired, two‐tailed Student's *t* test, *p < 0.05.

In airway diseases such as asthma, COPD and cystic fibrosis, the phenomenon of goblet cell hyperplasia is intimately associated with the extent of airway inflammation. To advance our understanding of the relationship between mucus production and inflammation within the context of COVID‐19, we explored in depth the inflammatory properties of these BSCs. For xenografts derived from control and COVID‐19 BSCs collected on Day 28, we conducted histological staining to ascertain their properties. Control xenografts predominantly comprised epithelial cells, forming cysts in an apically polarised manner, leaving significant vacant spaces within these cysts (Figures 2A and S2A). In contrast, COVID‐19 xenografts showed cysts populated by both epithelial and non‐epithelial cells. Previous HLA‐A immunohistochemistry staining revealed that these luminal non‐epithelial cells were not of human origin but of murine origin. Subsequent anti‐murine Ly6G staining on these cells identified a majority as neutrophils originating from NCG mice (Figures 2B and S2B). To quantify neutrophil infiltration, we categorised the levels of cysts into ‘None’, ‘Low’, ‘Medium’ and ‘High’ (Figure 2C). A significant increase in neutrophil infiltration was observed in COVID‐19 xenografts compared to control samples (Figures 2D and S2C), with ‘Medium’ and ‘High’ levels of neutrophil infiltration cysts prevailing (Figure S2D). This reflects the clinical manifestations of COVID‐19‐related airway inflammation, which exhibit similar patterns of neutrophil infiltration, underscoring the pivotal role of COVID‐19 BSCs in innate immunity. Given the compromised immune system of NCG mice, which lack T cells, NK cells, mature B cells and functional monocytes, our capacity to explore the nuances of immune cell recruitment beyond neutrophils was constrained. To address this, we harvested PBMCs from a healthy volunteer and activated them before stimulating with ALI condition medium derived from the control and COVID‐19 groups (Figures 2E and S2E). Within this adjusted experimental setup, we observed a noteworthy pattern: the COVID‐19 group exhibited an increased presence of IFN‐γ (+) CD4 and TNF‐α (+) CD4 cells compared to the control (Figure 2F,G). We further embarked on cytokine gene analysis to further explore the immune recruitment characteristics of COVID‐19 BSCs. In this analysis of COVID‐19 ALI—an in vitro, immune cell‐free niche for BSCs—we observed an upregulation in a broad array of cytokine genes. Chemokines associated with neutrophil recruitment, such as CXCL8, CXCL6, CXCL5, CXCL3 and CXCL1, were elevated, correlating with xenograft neutrophil infiltration and clinical observations. Additionally, chemokines related to lymphocyte activation, notably T helper cells, like CCL20, CXCL10 and CX3CL1, were also increased. Antiviral response signalling was examined, highlighting genes such as IFIT1, IF44L and MX1 that regulate airway antiviral responses. Furthermore, we identified a significant increase in interleukins and tumour necrosis factors, including IL‐1a, IL‐1b, IL‐6, IL‐17C, IL‐32, IL‐36G and TNF, along with its associated enzyme (Figure S3A). These factors are pivotal in modulating both innate and adaptive immune responses and may contribute to the airway inflammation loop, fostering a persistent inflammatory environment in the airway mucosa. This provides evidence for the clinical outcomes of COVID‐19, suggesting that beyond conventional antiviral responses, the observed upregulation of multiple cytokines related to diverse immune system activations indicates a hyperinflammatory state in COVID‐19 patients.

### 3.3 Pro‐Fibrotic Signatures in COVID‐19 BSCs

**FIGURE 3:**
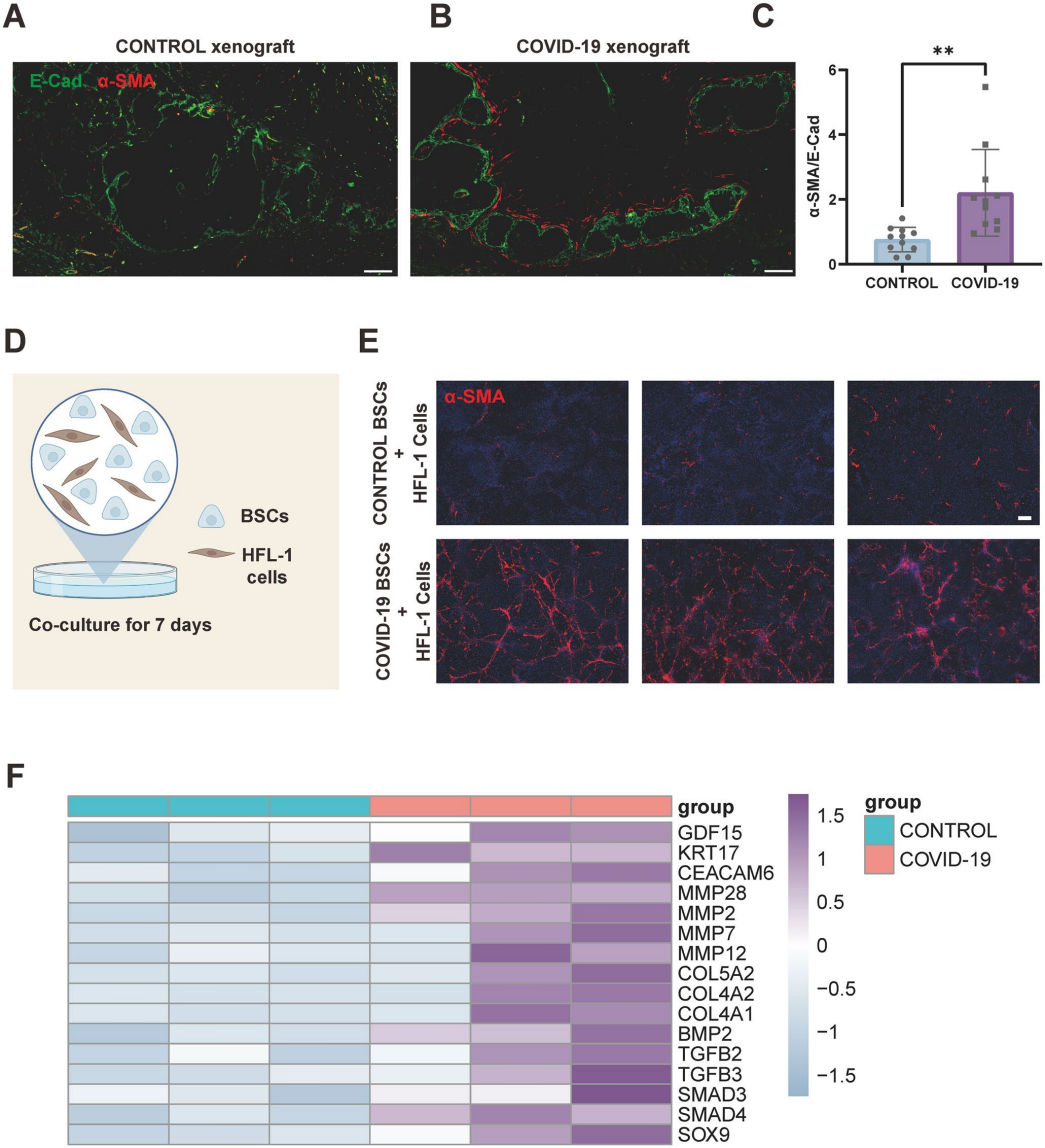
Fibrotic changes driven by COVID‐19 BSCs. (A) IF of E‐cadherin (green) and α‐SMA (red) imaging for control xenograft. Scale bar, 100 μm. (B) IF of E‐cadherin (green) and α‐SMA (red) imaging for COVID‐19 xenograft. Scale bar, 100 μm. (C) Quantification of α‐SMA/E‐Cad from control and COVID‐19 xenografts. Data are represented as the mean ± SD. *n* = 11, unpaired, two‐tailed Student's *t* test, **p < 0.01. (D)BSCs and HLF‐1 co‐culture assay. (E) IF images of HFL‐1 cells co‐cultured separately with control and COVID‐19 BSCs for 7 days, showing α‐SMA (red) (*n* = 3). Scale bar, 100 μm. (F) Heatmap comparing fibrosis gene expression in control and COVID‐19 ALI samples from bulk RNA‐seq data.

The post‐inflammatory pulmonary fibrosis following COVID‐19 is a significant concern, yet its underlying mechanisms remain poorly understood. Omics analyses have highlighted the proliferation of fibroblasts and the increased expression of fibrosis‐associated factors such as TGF‐β in COVID‐19‐affected lungs. In our investigation, we prominently noted the encasement of epithelial cysts by cells resembling fibroblasts within the COVID‐19 xenografts. Utilising antibodies targeting alpha smooth muscle actin (α‐SMA)—a crucial indicator of myofibroblast activation—we detected a significant alignment of these cells along the borders of the epithelial cysts in the COVID‐19 xenografts. This contrasted with the control xenografts, where such arrangements of myofibroblasts were markedly rarer around the cysts (Figures 3A,B and S3B,C). To assess the extent of fibrosis, we conducted a comparative analysis of the areas stained for α‐SMA, against those stained for E‐cadindicative of epithelial cells. Our findings demonstrated that the COVID‐19 xenografts exhibited considerably higher levels of fibrosis when compared to the control samples (Figure 3C). In our in vitro co‐culture experiments, human fetal lung fibroblasts were incubated with both control and COVID‐19 BSCs for 7 days. The COVID‐19 BSCs exhibited a pronounced increase in the myofibroblast marker α‐SMA (Figure 3D,E). The heightened expression of genes such as CEACAM6, KRT17 and GDF15 in COVID‐19 ALI models indicated a shift toward a pro‐fibrotic state in these BSCs. In addition, a spectrum of genes implicated in fibrosis repair, including COL5A2, COL4A2, COL4A1, MMP2, MMP7 and MMP12, showed elevated expression levels, pinpointing an activation of the TGFβ pathway alongside increases in factors such as SOX9, SMAD3, SMAD4 and TGFB2 (Figure 3F). These insights elucidate the possible mechanisms and pathways of COVID‐19‐associated pulmonary fibrosis, aligning closely with observed clinical manifestations in patients.

### 3.4 Transcriptomic Profiling of COVID‐19 BSCs

**FIGURE 4:**
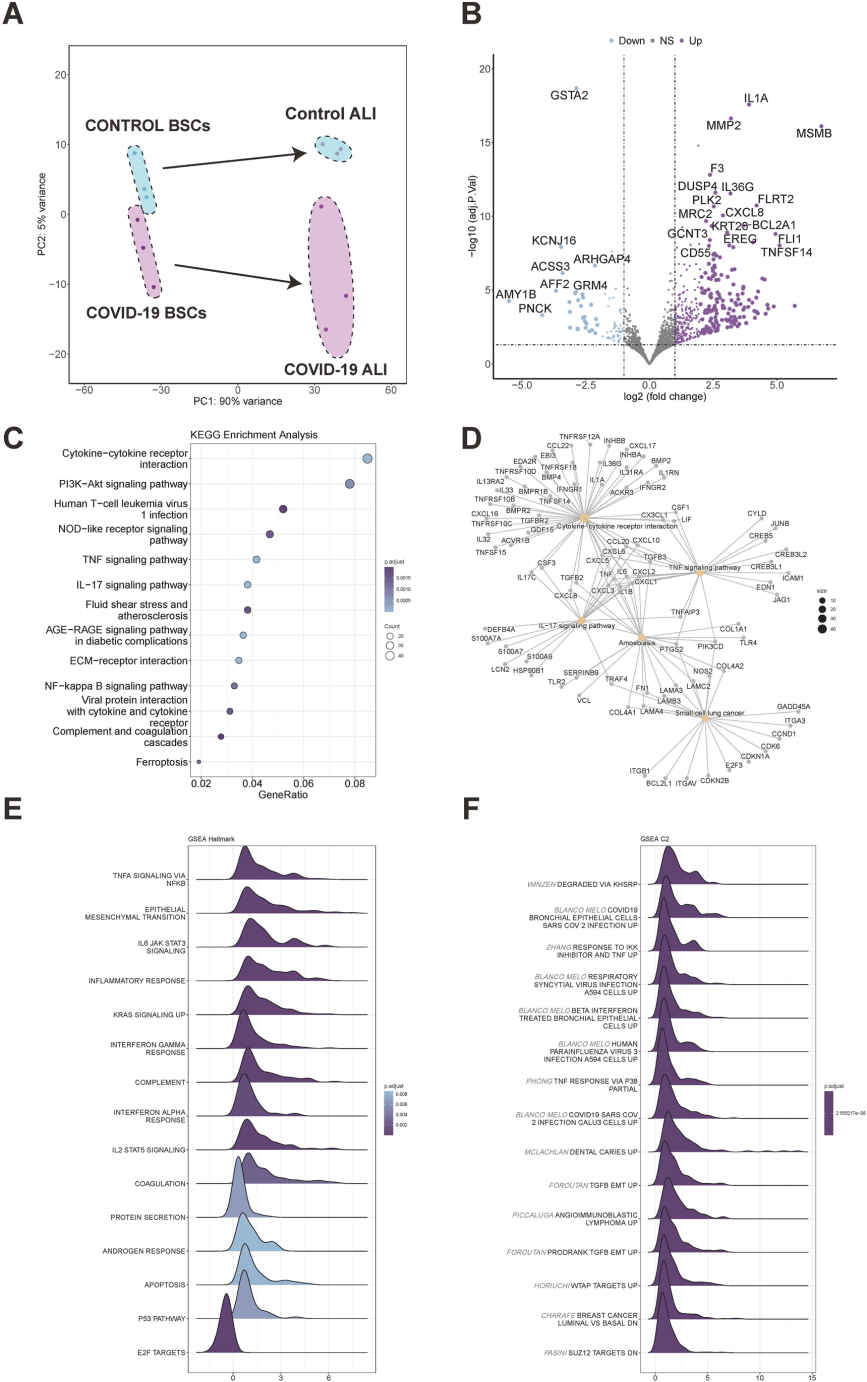
Transcriptomic landscapes of COVID‐19 BSCs. (A) PCA plot of bulk RNA‐seq data comparing COVID‐19 and control BSCs, control ALI and COVID‐19 ALI samples. (B) Volcano plot comparing the gene expression differences between COVID‐19 ALI and control ALI samples. (C) Dot plot of KEGG pathway enrichment analysis for COVID‐19 ALI versus control ALI samples. (D) Cnetplot visualisation highlighting enriched KEGG pathways in COVID‐19 ALI, with interconnected nodes representing key genes and their associated pathways. (E) Ridgeplot showcasing key Hallmark pathways in COVID‐19 ALI, underscoring pivotal biological processes and disease‐specific pathway activations. (F) Ridgeplot representation of GSEA C2 analysis within COVID‐19 ALI, detailing the involvement of curated gene sets from extensive biological databases and domain expertise.

Our research previously identified key aspects of differentiation, inflammation and fibrosis in COVID‐19 BSCs. To uncover the molecular basis of these findings, we conducted an in‐depth transcriptomic analysis of COVID‐19 BSCs. PCA analysis revealed distinct clustering between undifferentiated stem cells from COVID‐19 patients and controls, suggesting inherent transcriptional differences even before differentiation. Furthermore, differentiated BSCs from COVID‐19 patients and controls also clustered separately (Figure 4A), highlighting that these differences are further accentuated during differentiation. The volcano plot illustrates that genes implicated in inflammation and fibrosis, including IL1A, CXCL8 and MMP2, are significantly upregulated in COVID‐19 ALI. Concurrently, genes crucial for antioxidant production, such as GRM4 and GSTA2, are notably downregulated (Figure 4B). This pattern suggests a heightened oxidative stress and an extension of the stressed cellular state, underlining the severe disruption in cellular homeostasis induced by COVID‐19. Gene Ontology (GO) enrichment analysis revealed significant biological processes in COVID‐19 BSCs, including the positive regulation of cell adhesion, chemotaxis and wound healing (Figure S4A). Cellular component analysis indicated enrichment in cell‐substrate junctions, focal adhesion, collagen‐containing extracellular matrix and endoplasmic reticulum matrix (Figure S4B). Molecular function analysis highlighted cytokine activity, receptor binding, glycosaminoglycan binding, organic anion transmembrane transporter activity and sulfur compound binding (Figure S4C). These findings suggest that the severe inflammatory response triggered by COVID‐19 leads to enhanced migratory and adhesive properties of BSCs, potentially contributing to fibrosis. The enrichment of cellular components such as cell‐substrate junctions and collagen‐containing extracellular matrix supports this notion. Furthermore, the upregulation of cytokine activity and receptor binding indicates a robust inflammatory response, while alterations in glycosaminoglycan binding and transporter activity suggest significant impacts on cell signalling, metabolism and detoxification pathways. Further dissection through Kyoto Encyclopedia of Genes and Genomes (KEGG) pathway analysis revealed an enrichment of pathways pivotal in inflammatory and fibrotic processes, including PI3K‐AKT, TNF, IL‐17, AGE‐RAGE, NF‐kappa B and ECM receptor interactions, corroborating the transcriptomic data (Figure 4C). The Cnetplot places IL‐1b, TNF and IL‐6 as central nodes (Figure 4D), suggesting their integral roles in these pathways, echoing the cellular responses observed in single‐cell COVID‐19 epithelium and in vitro COVID‐19 exposed BSCs studies. Additionally, gene set enrichment analysis (GSEA) hallmark dataset validated these findings, highlighting enriched signalling of TNFA, IL6‐JAK‐STAT3, IL2‐STAT5, interferon‐γ response, interferon‐α response and epithelial–mesenchymal transition, all positively correlating with COVID‐19 BSCs pathway (Figure 4E). These results delineate a cascade of biological processes initiated by viral infection, leading to varied inflammatory responses. Consequently, these inflammatory responses exert a profound influence on BSCs differentiation, manifesting in a predisposition toward increased mucus secretion and contributing to the development of airway fibrotic damage. Surprisingly, the C2 curated gene set analysis through GSEA paralleled SARS‐CoV‐2 infected epithelium, suggesting our COVID‐19 BSCs can exhibit both viral response patterns and possible diseased phenotype autonomously without the infection of SARS‐CoV‐2 (Figure 4F). This layered analysis accentuates our COVID‐19 BSCs' innate capacity to replicate viral response pathways and related disease patterns, a discovery that significantly propels the potential utility of our models in dissecting the intricate mechanisms and pathophysiology underlying COVID‐19.

### 3.5 MSCs Mediate Phenotypic Mitigation in COVID‐19 BSCs

**FIGURE 5:**
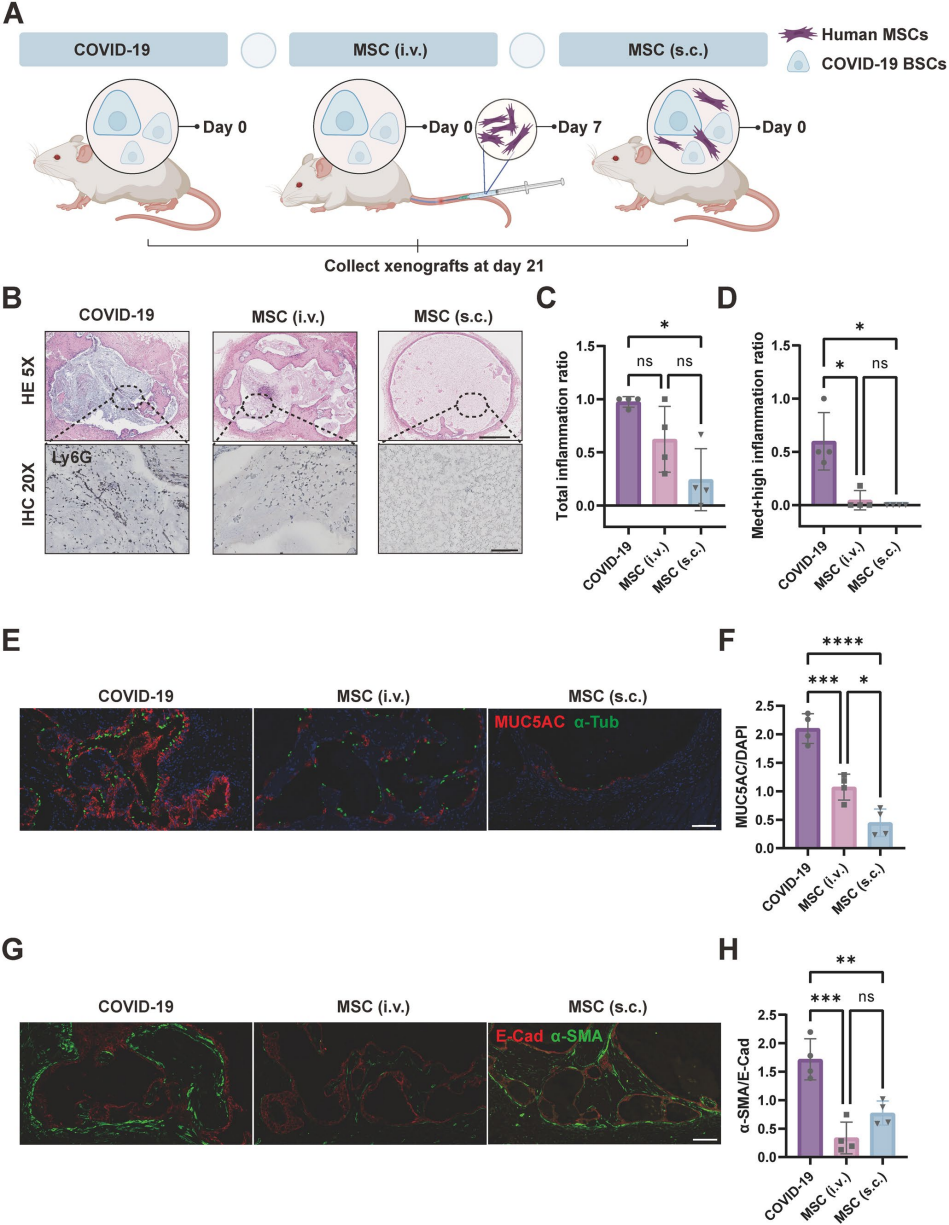
MSCs in Counteracting Phenotypic Deviations in COVID‐19 BSCs. (A) The visual outlines detail two experimental designs for in vivo co‐culture assays of COVID‐19 BSCs with MSC. (B) Top: H&E staining of COVID‐19, MSC (i.v.) and MSC (s.c.) xenografts. Scale bar, 500 μm. Bottom: IHC of Ly6G (brown) for COVID‐19, MSC (i.v.) and MSC (s.c.) xenografts. Scale bar, 100 μm. (C) Quantification of total inflammation infiltration ratio from COVID‐19, MSC (i.v.) and MSC (s.c.) xenografts. Data are represented as the mean ± SD. *n* = 4, Kruskal–Wallis *H* test, Dunn's post hoc test, ns = not significant, *p < 0.05. (D) Quantification of medium and high inflammation infiltration ratio from COVID‐19, MSC (i.v.) and MSC (s.c.) xenografts. Data are represented as the mean ± SD. *n* = 4, Kruskal–Wallis *H* test, Dunn's post hoc test, ns = not significant, *p < 0.05. (E) IF of MUC5AC (red) and α‐Tub (green) imaging for COVID‐19, MSC (i.v.) and MSC (s.c.) xenografts. Scale bar, 100 μm. (F) Quantification of MUC5AC+ cells from COVID‐19, MSC (i.v.) and MSC (s.c.) xenografts epithelial cysts. Data are represented as the mean ± SD. *n* = 4, ordinary one‐way ANOVA, Tukey's multiple comparisons test, *p < 0.05, ***p < 0.001, ****p < 0.0001. (G) IF of α‐SMA (green) and E‐cadherin (red) imaging for COVID‐19, MSC (i.v.) and MSC (s.c.) xenografts. Scale bar, 100 μm. (H) Quantification of α‐SMA/E‐Cad from COVID‐19, MSC (i.v.) and MSC (s.c.) xenografts epithelial cysts. Data are represented as the mean ± SD. *n* = 4, ordinary one‐way ANOVA, Tukey's multiple comparisons test, ns = not significant, **p < 0.01, ***p < 0.001.

Building on our observations of COVID‐19 BSCs displaying phenotypes of inflammation, excessive mucus secretion and fibrosis, we sought to identify an effective treatment modality. MSCs, renowned for their immune‐modulatory and regenerative properties, emerged as a promising therapeutic candidate. Accordingly, we initiated in vivo experiments to evaluate MSC efficacy. Our methodology entailed two distinct in vivo co‐culture assays involving COVID‐19 BSCs and MSCs. In the COVID‐19 model, we administered an initial subcutaneous injection of COVID‐19 BSCs. For the MSC intravenous (i.v.) group, additional MSC injections were introduced intravenously on Day 7 into NCG mice. Alternatively, the MSC subcutaneous (s.c.) group received a one‐time co‐injection of COVID‐19 BSCs and MSCs subcutaneously, simulating various clinical MSC administration routes to patients, either through intravenous injection or local airway delivery. By Day 21, nodules from both experimental groups underwent histological assessments and immunostaining analyses (Figures 5A and S5A). In the MSC (i.v.) group, there was a reduction in overall inflammation compared to the COVID‐19 group, but the difference did not reach statistical significance (Figure 5B,C). This group, however, showed a significant decrease in the levels of medium to high inflammation (Figure 5D). The MSC (s.c.) group also indicated a decrease in both overall inflammation and in medium to high levels of inflammation (Figure 5C,D). A comparable trend was noted in mucus hyperplasia, with both MSC (i.v.) and MSC (s.c.) groups showing a reduction in MUC5AC‐positive cells within epithelial nodules (Figures 5E,F and S5B). Notably, the decrease in the MSC (s.c.) group was more pronounced. Further examination of fibrosis properties in these groups showed that both MSC treatments lessened myofibroblast activation, with the intravenous MSC administration being notably effective (Figure 5G,H). Complementary in vitro studies co‐culturing COVID‐19 BSCs and MSCs in an ALI setup revealed downregulation of genes associated with the interferon pathway, including IFIT2, IFI6, IFIT3, ISG15, IFI44L, IFIT1 and IFI44, suggesting that MSCs could act as anti‐inflammatory agents (Figure S5C). This indicates their potential in mitigating COVID‐19 symptoms by modulating antiviral pathways, positioning MSC therapy as a viable option for patients with prolonged COVID‐19 symptoms experiencing chronic inflammation.

## 4 Discussion

Malfunctioning BSCs provide new insights into the pathology of COVID‐19 and PASC, showing that their increased goblet cell hyperplasia, pro‐inflammatory and pro‐fibrotic tendencies are self‐sustained. MSCs emerge as promising agents capable of alleviating these pathological states in COVID‐19 BSCs, presenting an innovative path for therapeutic strategies. Our research highlights three key findings: firstly, BSCs associated with COVID‐19 inherently display pathological phenotypes without external provocation. Secondly, these pathologies are reversible, suggesting potential for recovery from their disease‐driven states. Lastly, MSCs present a practical treatment option, aiming to ameliorate the detrimental effects observed in BSCs due to COVID‐19.

In our study, utilising ALI and xenograft models, we observed elevated ACE2 expression in BSCs from COVID‐19 patients, the primary receptor for SARS‐CoV‐2 and a key factor linked to disease severity [25]. Additionally, BSCs from COVID‐19 patients demonstrated a propensity for goblet cell differentiation, indicated by the upsurge of MUC5AC‐positive cells. Gene expression analysis confirmed this, showing elevated levels of mucus‐related genes such as MUC5B, SCGB1A1, SCGB3A1 and associated transcription factors. A study focusing on in vitro differentiation of COVID‐19 BSCs also reported increased MUC5AC expression [26]. However, it did not detect a rise in MUC5B, suggesting potential IL‐1B signalling pathway deficits. This disparity may be attributable to the airway region from which the BSCs were derived. The comparison study, using cells from tracheal aspirates, likely sampled BSCs from smaller bronchi, indicating that cells from various airway levels might respond differently to inflammation. Additionally, our findings offer a distinct contrast to the in vitro SARS‐CoV‐2 infection models utilising BSCs from healthy volunteers under ALI conditions. Contrary to the mucous differentiation observed in BSCs derived from COVID‐19 patients, these models did not report a significant increase in goblet cell proliferation [27, 28, 29]. Interestingly, the inflammatory responses elicited in these in vitro settings substantially diverged from those noted in BSCs obtained from patients, especially with the marked absence of IL‐1 pathway activation [29]. This observation indicates that the intricate process of mucous differentiation in BSCs likely requires the mediation of immune or mesenchymal cells present within the patient's airway environment, suggesting that BSCs isolated from patients retain a ‘memory’ of their interactions with these cells rather than the virus itself.

Our observations also revealed that COVID‐19 BSCs exhibit strong inflammatory recruitment characteristics, along with pro‐fibrotic capabilities, mirroring the clustering of inflammation observed in the airways of COVID‐19 patients. We notice key mediators such as TNF‐α, IL‐6, IL‐1, interferon‐α, interferon‐γ and the fibrosis‐related TGF‐β playing significant roles in COVID‐19 BSCs molecular level. Identifying the relationship between these pathways and the prognosis of COVID‐19 patients could be instrumental in devising management strategies. Intriguingly, similar inflammatory and pro‐fibrotic recruitment capabilities have been observed in BSCs from patients with COPD [19, 30] and IPF [18], as well as through genomic sequencing of COVID‐19 patients [10, 11]. Our research lays the groundwork for further investigation into respiratory complications of PASC and potential interventions.

To further understand the dynamic interactions between immune cells and BSCs in COVID‐19 and overcome the limitations of studying immune responses in NCG mice, we co‐cultured activated PBMCs from a healthy volunteer with ALI condition medium. Previous studies have shown that both IFN‐γ‐producing and TNF‐α‐producing CD4+ T cells are essential in the immune response to COVID‐19 [31, 32]. In our co‐culture experiment, we observed a significant increase in IFN‐γ (+) CD4 and TNF‐α (+) CD4 cells in the COVID‐19 group compared to control. This suggests that BSCs play a crucial role in priming adaptive immune cells, especially CD4+ T cells. Beyond adaptive immunity, the innate immune system also plays a critical role in shaping the airway's response to SARS‐CoV‐2 infection. In addition to the neutrophil infiltration observed in our experiments, recent studies using human pluripotent stem cell‐derived airway and macrophage co‐culture systems demonstrated the therapeutic potential of modulating macrophage polarisation and blocking viral entry to protect lung cells from SARS‐CoV‐2‐induced damage [33]. These complementary findings highlight the interplay between immune cells and epithelial cells, further emphasising the need to explore targeted interventions that address both epithelial and immune‐mediated pathways in COVID‐19.

Within our investigations, MSCs displayed varied effects: notably mitigating the COVID‐19 BSCs disease phenotypes during in vivo assessments. In vitro analyses indicated focused modulation of the interferon pathway without impacting other phenotype‐related genes, underscoring the complex and systemic nature of MSC therapy. Unlike static in vitro conditions, the dynamic in vivo environment allows MSCs to interact with multiple biological systems, enhancing their therapeutic potential through immune modulation and interactions with the extracellular matrix and tissue cells. Previous studies have shown that MSC therapy provides long‐term benefits in severe COVID‐19 patients, including improved lung lesion recovery and symptom resolution [34]. Complementing these observations, MSC therapy has also been shown to enhance DNA repair pathways in critically ill COVID‐19 patients [35]. Additionally, RAP1, a DNA repair molecule, has been linked to improved cell survival under stress through reduced NF‐κB activation [36]. Together, these studies underscore the interconnected roles of RAP1‐NF‐κB‐IFN‐γ signalling and DNA repair in the therapeutic potential of MSCs for COVID‐19‐related complications.

Our study provides foundational insights that advance the clinical translation of MSC therapies. MSC‐based therapies offer a promising approach for treating COVID‐19 and PASC‐related respiratory complications. The latest models such as patient‐specific xenografts and organoid co‐culture systems provide innovative platforms to investigate MSC dosages and their interactions within diverse cellular environments. These models enable the assessment of different diseases' and patients' sensitivities to MSC therapy, dose–response relationships and long‐term therapeutic outcomes, facilitating the development of personalised stem cell treatments. Although MSCs have been applied in clinical settings, scaling up their production for widespread use presents significant challenges [22]. Robust biomanufacturing processes, including the use of bioreactors and cryopreservation protocols aligned with good manufacturing practices [37], are essential to ensure consistent cell quality and therapeutic potency. Additionally, optimising MSC dosage is critical to maximise efficacy and minimise risks. Long‐term safety and efficacy are paramount for the successful integration of MSC therapies into clinical practice. Current research is evaluating the sustained effects and potential adverse events associated with MSC administration, providing evidence to support their clinical relevance [38]. By addressing these challenges of scalability, optimal dosing and ensuring long‐term safety, MSCs can be more effectively integrated into mainstream clinical practice.

Our study has limitations primarily related to the inherent constraints of experimental design. While our model provides valuable insights, it may not encompass all the environmental and genetic factors influencing BSCs behaviour. Future research will aim to incorporate more comprehensive environmental data to build on our findings.

## 5 Conclusion

COVID‐19 BSCs demonstrated pronounced goblet cell hyperplasia, inflammatory responses and tendencies toward fibrosis. Co‐culture with MSCs notably reduced these pathological changes, likely due to MSCs' regulatory effects on the interferon signalling pathway.

## Author Contributions

S.L., X.S. and C.J. contributed to the study design. C.T., Z.S., L.W. and S.D. contributed to patient recruitment and sample collection. S.D., X.C., M.Z., J.W. carried out the cell and animal experiments. S.D. and Y.Z. performed the statistical analysis. S.D. and J.J. conducted the bioinformatics analysis. S.D. and X.S. drafted the manuscript. All authors contributed to critical manuscript revision and provided approval to submit the manuscript for publication.

## Ethics Statement

This study was approved by the hospital's Ethics Committee (Ethics ID: 2022138) and conducted in accordance with the Declaration of Helsinki. Informed consent was obtained from all participants.

## Conflicts of Interest

The authors declare no conflicts of interest.

## Supporting information


**Figure S1.** (A) Top: H&E staining of control (*n* = 4) and COVID‐19 (*n* = 4) xenografts. Scale bar, 100 μm. Bottom: IF of MUC5AC (green) and α‐Tub (red) imaging for control xenografts (*n* = 4). Scale bar, 100 μm. (B) Top: H&E staining of control (*n* = 4) and COVID‐19 (*n* = 4) xenografts. Scale bar, 100 μm. Bottom: IF of MUC5AC (green) and α‐Tub (red) imaging for COVID‐19 xenografts (*n* = 4). (C) Heatmap of differential gene expression in airway epithelial cells, comparing control and COVID‐19 ALI samples from bulk RNA‐seq data.

**Figure S2.** (A) H&E staining of control (*n* = 4) xenografts. Scale bar, 100 μm. (B) H&E staining of COVID‐19 (*n* = 4) xenografts. Scale bar, 100 μm. (C) Quantification of total inflammation infiltration ratio from control and COVID‐19 xenografts. Data are represented as the mean ± SD. *n* = 11, non‐parametric *t* test, ****p < 0.0001. (D) Quantification of medium and high inflammation infiltration ratio from control and COVID‐19 xenografts. Data are represented as the mean ± SD. *n* = 11, non‐parametric *t* test, ****p < 0.0001. (E) Flow cytometry gating strategy for PBMC subsets.

**Figure S3.** (A) Heatmap showcasing cytokine gene expression differences between control and COVID‐19 ALI samples from bulk RNA‐seq data. (B) IF of E‐cadherin (green) and α‐SMA (red) imaging for control (*n* = 4) xenografts. Scale bar, 100 μm. (C) IF of E‐cadherin (green) and α‐SMA (red) imaging for COVID‐19 (*n* = 4) xenografts. Scale bar, 100 μm.

**Figure S4.** (A) Dot plot representing Gene Ontology (GO) enrichment analysis across COVID‐19 ALI compared to control ALI samples, showcasing disparities in biological processes. (B) Dot plot representing GO enrichment analysis across COVID‐19 ALI compared to control ALI samples, showcasing disparities in cellular components. (C) Dot plot representing GO enrichment analysis across COVID‐19 ALI compared to control ALI samples, showcasing disparities in molecular functions.

**Figure S5.** (A) Xenografts formed by each group. (B) H&E staining of xenografts. Scale bar, 100 μm. (C) Heatmap of bulk RNA‐seq data displaying differential expression between COVID‐19 ALI samples and those co‐cultured with MSCs.

**Table S1.** Clinical Characteristics of the COVID‐19 Patients and the control individuals.**Table S2.** Clinical Characteristics of the COVID‐19 Patients and the control individuals.

## Data Availability

The data that support the findings of this study are available from the corresponding author upon reasonable request.
