# Supplementary figures and images for: Airway Basal Stem Cells Inflammatory Alterations in COVID‐19 and Mitigation by Mesenchymal Stem Cells
Source: Cell Prolif. 2025 Jan 26;58(6):e13812. doi: 10.1111/cpr.13812 (PMC12179551; doi:10.1111/cpr.13812)

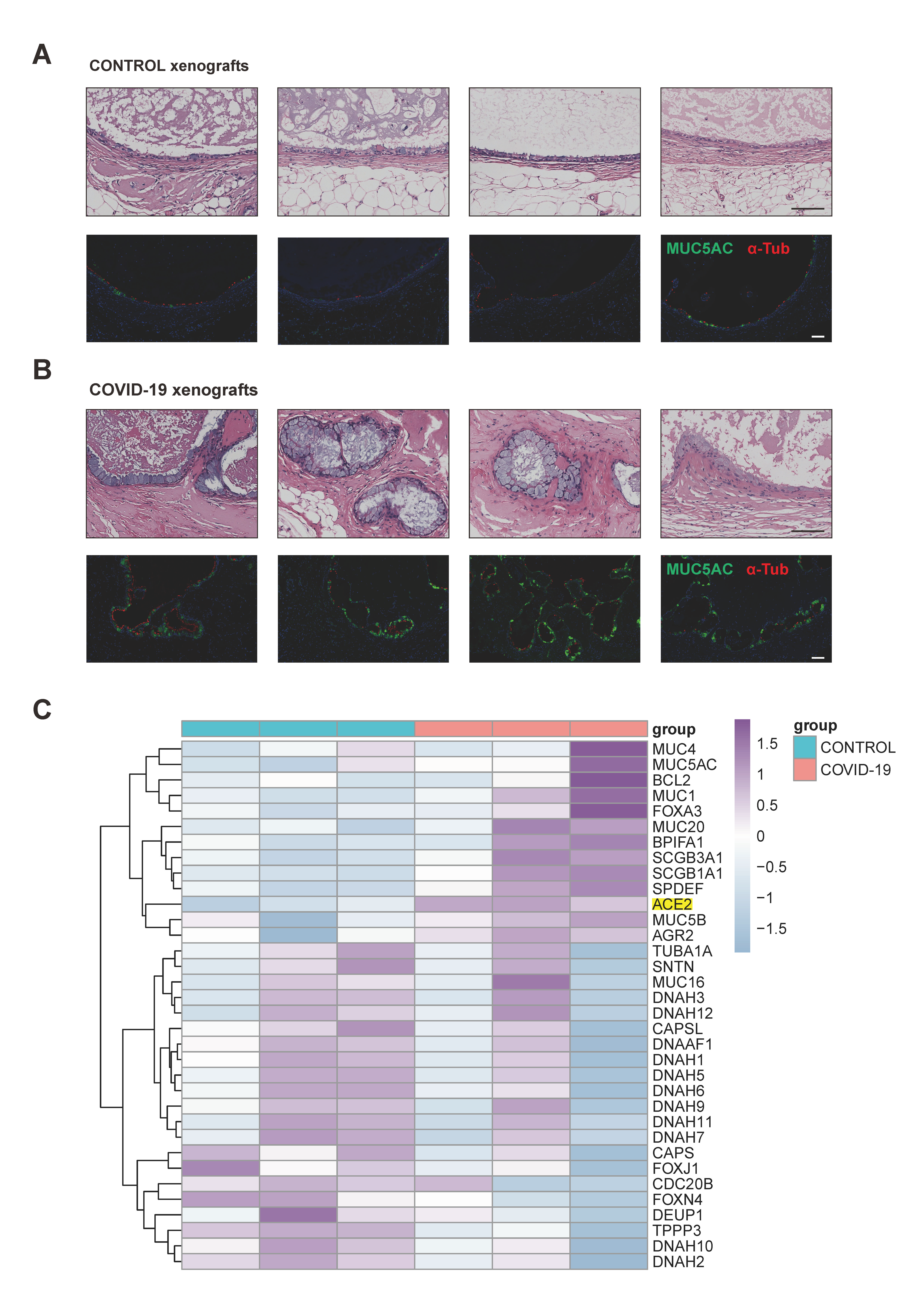

Supplement: Supplementary file 1 — Figure S1. (A) Top: H&E staining of control (n = 4) and COVID‐19 (n = 4) xenografts. Scale bar, 100 μm. Bottom: IF of MUC5AC (green) and α‐Tub (red) imaging for control xenografts (n = 4). Scale bar, 100 μm. (B) Top: H&E staining of control (n = 4) and COVID‐19 (n = 4) xenografts. Scale bar, 100 μm. Bottom: IF of MUC5AC (green) and α‐Tub (red) imaging for COVID‐19 xenografts (n = 4). (C) Heatmap of differential gene expression in airway epithelial cells, comparing control and COVID‐19 ALI samples from bulk RNA‐seq data. [file CPR-58-e13812-s001.tif]

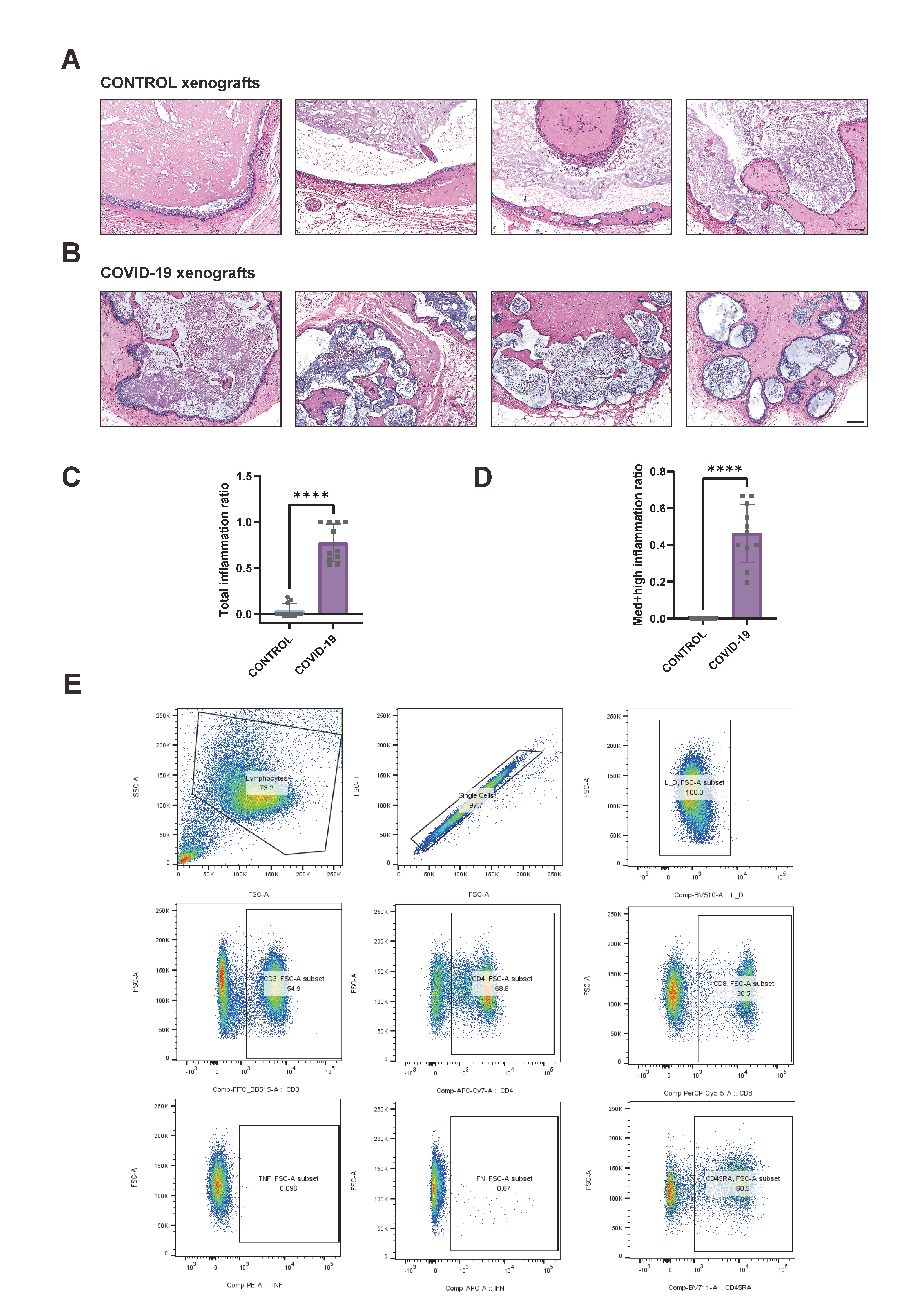

Supplement: Supplementary file 2 — Figure S2. (A) H&E staining of control (n = 4) xenografts. Scale bar, 100 μm. (B) H&E staining of COVID‐19 (n = 4) xenografts. Scale bar, 100 μm. (C) Quantification of total inflammation infiltration ratio from control and COVID‐19 xenografts. Data are represented as the mean ± SD. n = 11, non‐parametric t test, ****p < 0.0001. (D) Quantification of medium and high inflammation infiltration ratio from control and COVID‐19 xenografts. Data are represented as the mean ± SD. n = 11, non‐parametric t test, ****p < 0.0001. (E) Flow cytometry gating strategy for PBMC subsets. [file CPR-58-e13812-s006.tif]

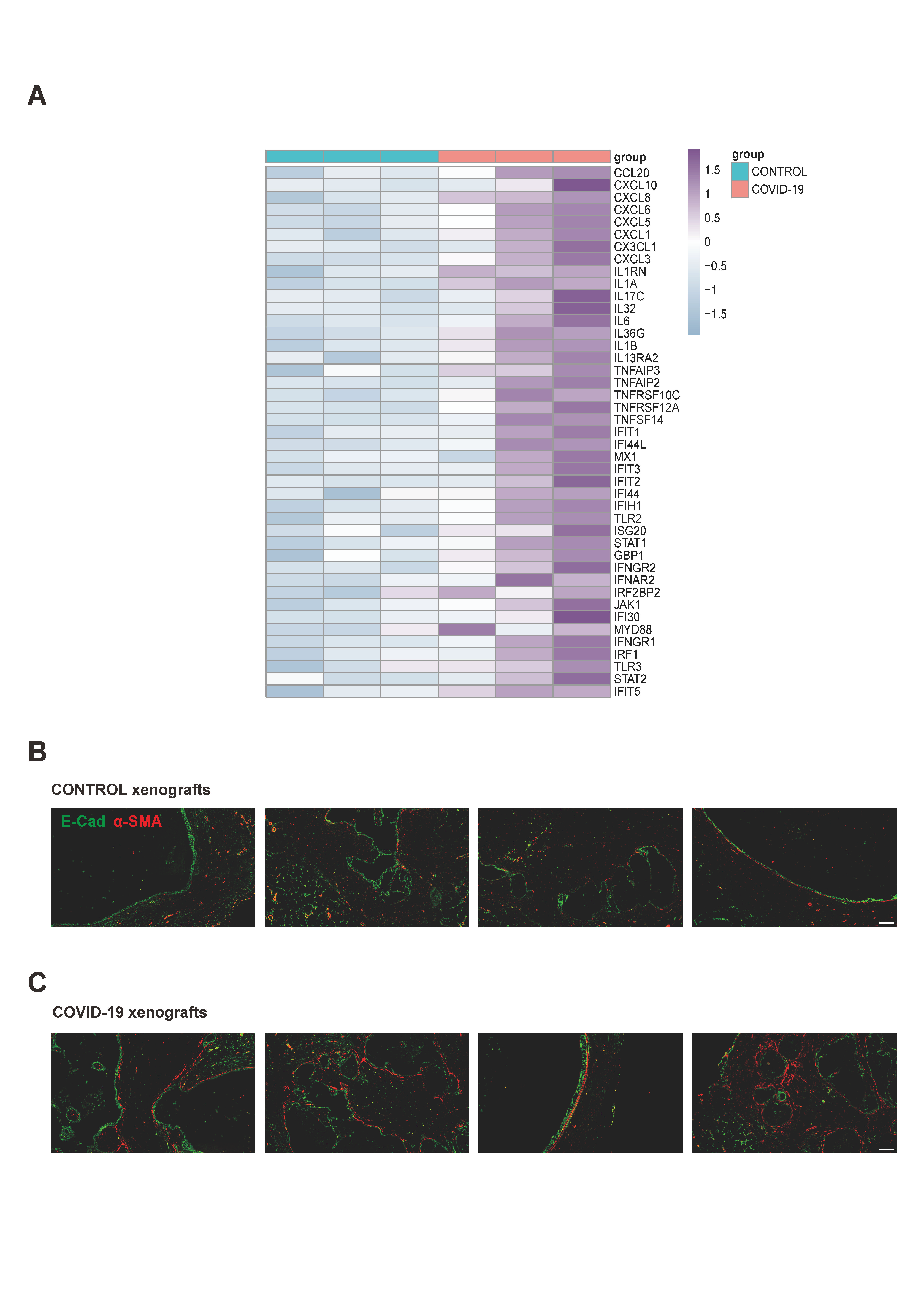

Supplement: Supplementary file 3 — Figure S3. (A) Heatmap showcasing cytokine gene expression differences between control and COVID‐19 ALI samples from bulk RNA‐seq data. (B) IF of E‐cadherin (green) and α‐SMA (red) imaging for control (n = 4) xenografts. Scale bar, 100 μm. (C) IF of E‐cadherin (green) and α‐SMA (red) imaging for COVID‐19 (n = 4) xenografts. Scale bar, 100 μm. [file CPR-58-e13812-s004.tif]

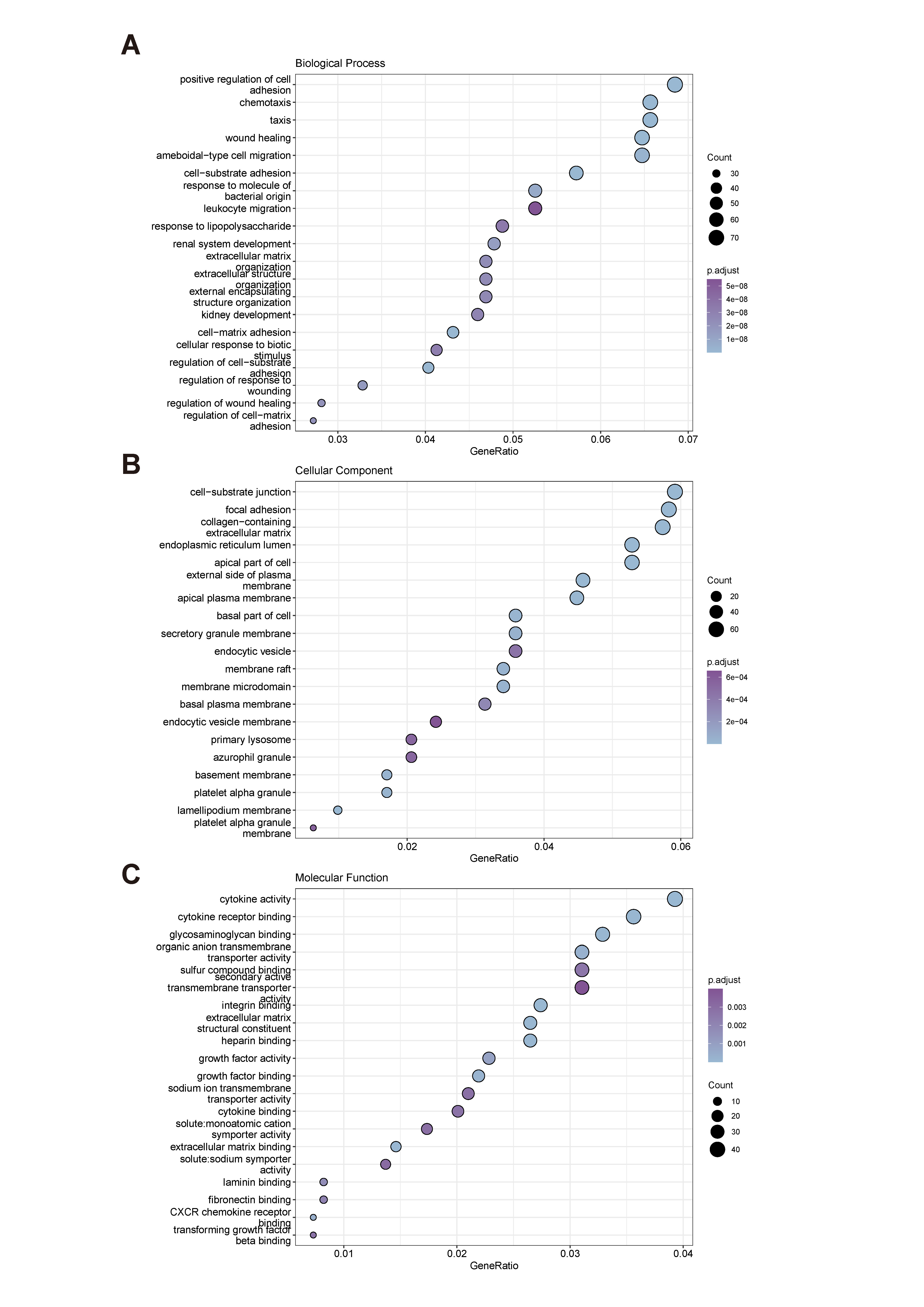

Supplement: Supplementary file 4 — Figure S4. (A) Dot plot representing Gene Ontology (GO) enrichment analysis across COVID‐19 ALI compared to control ALI samples, showcasing disparities in biological processes. (B) Dot plot representing GO enrichment analysis across COVID‐19 ALI compared to control ALI samples, showcasing disparities in cellular components. (C) Dot plot representing GO enrichment analysis across COVID‐19 ALI compared to control ALI samples, showcasing disparities in molecular functions. [file CPR-58-e13812-s002.tif]

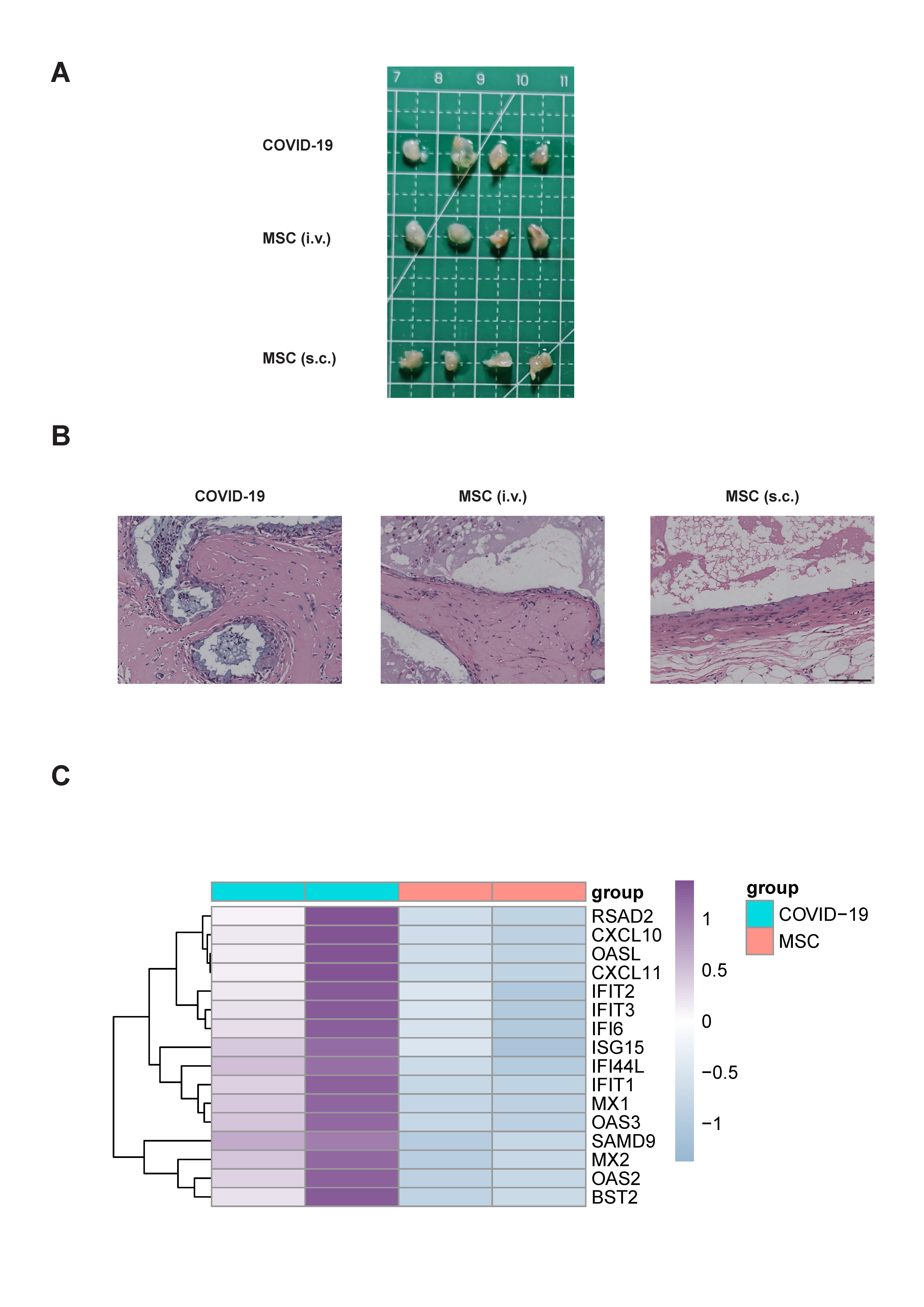

Supplement: Supplementary file 5 — Figure S5. (A) Xenografts formed by each group. (B) H&E staining of xenografts. Scale bar, 100 μm. (C) Heatmap of bulk RNA‐seq data displaying differential expression between COVID‐19 ALI samples and those co‐cultured with MSCs. [file CPR-58-e13812-s005.tif]
