# Supplementary material for: Airway Basal Stem Cells Inflammatory Alterations in COVID‐19 and Mitigation by Mesenchymal Stem Cells
Source: Cell Prolif. 2025 Jan 26;58(6):e13812. doi: 10.1111/cpr.13812 (PMC12179551; doi:10.1111/cpr.13812)
Supplement: Supplementary file 6 — Table S1. Clinical Characteristics of the COVID‐19 Patients and the control individuals.Table S2. Clinical Characteristics of the COVID‐19 Patients and the control individuals. [file CPR-58-e13812-s003.docx]

Table S1. Clinical Characteristics of the COVID-19 Patients and the control individuals

|  | COVID-19 | CONTROL |
| --- | --- | --- |
| n | 11 | 11 |
| Age — yr | 58.73±14.06 | 44.36±14.60 |
| Female sex — no. (%) | 4 (36.36) | 6 (54.55) |
| Admission to Sample Time- days | 4.72±2.33 | \ |
| Mild to Moderate COVID-19— no. (%) | 8 (72.72) | \ |
| Severe COVID-19— no. (%) | 3 (27.27) | \ |

Table S2. Clinical Characteristics of the COVID-19 Patients and the control individuals

| Study ID | Age | Sex | Lung Diseases | Additional Conditions |
| --- | --- | --- | --- | --- |
| COVID-19-1 | 50 | F | COVID-19 | LTX |
| COVID-19-2 | 72 | M | COVID-19 | LTX |
| COVID-19-3 | 63 | M | COVID-19 | LTX |
| COVID-19-4 | 46 | F | COVID-19 | LTX |
| COVID-19-5 | 64 | M | COVID-19 | LTX |
| COVID-19-6 | 65 | F | COVID-19 | LTX |
| COVID-19-7 | 57 | M | COVID-19 | LTX |
| COVID-19-8 | 81 | M | COVID-19, COPD |  |
| COVID-19-9 | 33 | F | COVID-19 |  |
| COVID-19-10 | 70 | M | COVID-19, Asthma |  |
| COVID-19-11 | 45 | M | COVID-19 |  |
| CONTROL-1 | 33 | F | None | LTX |
| CONTROL-2 | 54 | F | None | LTX |
| CONTROL-3 | 64 | M | None | LTX |
| CONTROL-4 | 39 | M | None | LTX |
| CONTROL-5 | 48 | M | None | LTX |
| CONTROL-6 | 20 | M | None |  |
| CONTROL-7 | 40 | M | None |  |
| CONTROL-8 | 35 | F | None |  |
| CONTROL-9 | 33 | F | None |  |
| CONTROL-10 | 54 | F | None | LTX |
| CONTROL-11 | 68 | F | None |  |

Abbreviations: F: Female; M: Male; LTX: Lung Transplant; COPD: Chronic Obstructive Pulmonary Disease
